# Activities of the Novel Bacterial Topoisomerase Inhibitor OSUAB-0284 against the Biothreat Pathogen *Bacillus anthracis* and Its Type II Topoisomerases

**DOI:** 10.1021/acsinfecdis.5c00860

**Published:** 2025-12-23

**Authors:** Chelsea A. Mann, Stephanie A. Halasohoris, Annette M. Gray, Jennifer Chua, Jade L. Spencer, Bobby J. Curry, Ashley L. Babyak, Christopher P. Klimko, Christopher K. Cote, Jason S. West, Mark J. Mitton-Fry, J. Matthew Meinig, Neil Osheroff

**Affiliations:** † Department of Biochemistry, 12327 Vanderbilt University School of Medicine, Nashville, Tennessee 37232, United States; ‡ Bacteriology Division, 70051 United States Army Medical Research Institute of Infectious Diseases, Frederick, Fort Detrick, Maryland 21702, United States; § Division of Medicinal Chemistry and Pharmacognosy, 15480 The Ohio State University College of Pharmacy, Columbus, Ohio 43210, United States; ∥ Departments of Biochemistry and Medicine (Hematology/Oncology), Vanderbilt University School of Medicine, Nashville, Tennessee 37232, United States

**Keywords:** OSUAB-0284, gepotidacin, gyrase, topoisomerase IV, *Bacillus anthracis*, anthrax

## Abstract

*Bacillus anthracis* is the etiological agent of anthrax and is classified as a Tier 1 biothreat pathogen. The fluoroquinolone ciprofloxacin is a preferred prophylactic drug for potential anthrax infections and acts by stabilizing DNA strand breaks formed by the bacterial type II topoisomerases, gyrase and topoisomerase IV. Unfortunately, widespread fluoroquinolone usage has increased levels of resistance in common bacterial pathogens, raising concern that resistant *B. anthracis* strains could be misused. Therefore, there is great interest in developing new classes of antibacterials that are efficacious against both wild-type and fluoroquinolone-resistant *B. anthracis* infections. Previous studies have demonstrated that gepotidacin, a triazaacenaphthylene antibacterial that targets gyrase and topoisomerase IV, displays potent activity against *B. anthracis* and was efficacious in a rabbit inhalation anthrax model. Given these promising results, we evaluated the activity of OSUAB-0284, a Novel Bacterial Topoisomerase Inhibitor (NBTI) that shares a general pharmacophore with gepotidacin, against *B. anthracis*. OSUAB-0284 displayed activity against *B. anthracis* that was comparable to or better than gepotidacin. Both compounds displayed activity against fluoroquinolone-resistant cells. Gepotidacin and OSUAB-0284 increased levels of gyrase- and topoisomerase IV-mediated DNA single-stranded breaks and inhibited the overall catalytic activity of the two enzymes. Both compounds were also more potent than ciprofloxacin against wild-type gyrase and topoisomerase IV and maintained activity against fluoroquinolone-resistant enzymes. Finally, OSUAB-0284 displayed efficacy in a mouse model of inhalation anthrax. These results provide mechanistic underpinnings supporting the use of gepotidacin and OSUAB-0284 against *B. anthracis* and suggest that they may be potential candidates for the treatment of anthrax.

## Graphical abstract

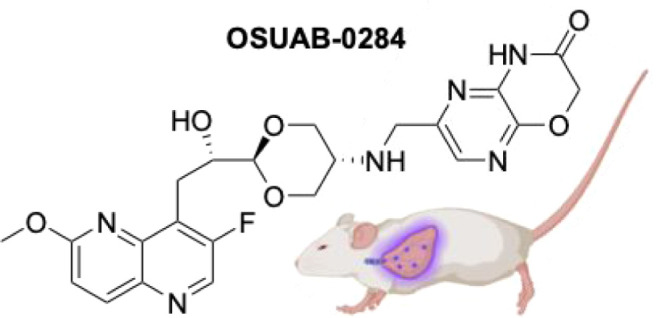

## Introduction

*Bacillus anthracis* is the causative agent of anthrax.^1,2^ It is a spore-forming soil bacterium that becomes metabolically active when taken up by its host. Anthrax is an ancient disease that predates the biblical era.^3^ Historically, the cutaneous infection is the most common and is associated with wool handlers.^3^ The name anthrax, which is Greek for coal, was coined by Hippocrates and is derived from the anthracene-colored sores associated with the physical presentation of the cutaneous disease.^4^ Anthrax can also manifest in the gastrointestinal tract due to ingestion of the bacterium or in the lungs through inhalation of *B. anthracis* spores.^5^ Of the disease forms, inhalation anthrax is by far the most lethal, with estimates ranging from 45 to 75% mortality even with treatment. In the absence of treatment, it is nearly 100% fatal.^5^

Anthrax holds a unique place in medical history as the infection that was chiefly responsible for the development of Koch’s germ theory of disease and Pasteur’s early work on vaccines.^3,4^ However, in modern times, *B. anthracis* is more well-known for its role as a biothreat pathogen.^6,7^ As an example, in 2001, a series of letters that contained anthrax spores were mailed to U.S. news media and government officials, including members of congress and federal judges.^8^ Thousands of civilians were potentially exposed to the pathogen and unfortunately, 22 people contracted anthrax, which led to five fatalities.^8^ In the immediate aftermath of this attack, sales of ciprofloxacin (Figure 1), a fluoroquinolone that is the primary drug used for anthrax prophylaxis,^9^ spiked by several thousand prescriptions per day.^7,10^ Furthermore, the attack prompted the U.S. government to negotiate for the purchase of 100 million doses of the antibacterial in case of future attacks.^11^

**Figure 1:**
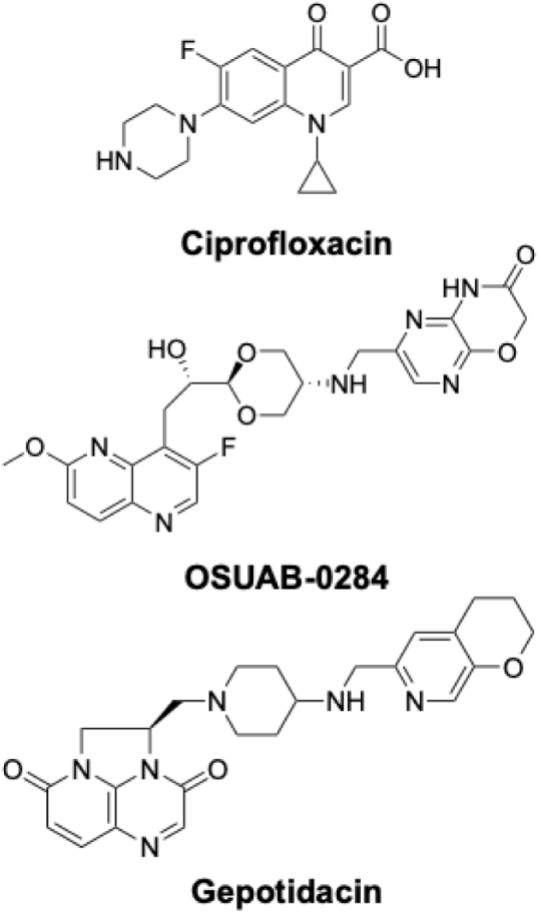
Structures of the fluoroquinolone ciprofloxacin, NBTI OSUAB-0284, and triazaacenaphthylene gepotidacin.

The primary targets for ciprofloxacin are the bacterial type II topoisomerases gyrase and topoisomerase IV.^12–15^ These essential enzymes regulate levels of DNA under and overwinding and remove knots and tangles from the bacterial chromosome, respectively. Gyrase and topoisomerase IV perform their essential cellular functions by passing an intact double helix through a transient double-stranded break that they generate in another segment of DNA.^12,13,15–18^ To maintain genomic integrity during this process, the enzymes form covalent bonds between active site tyrosine residues and the phosphate moieties at the newly generated 5′-DNA termini on the two strands.^16–18^ This covalent complex between the enzyme and cleaved DNA is known as the cleavage complex.^12,16^

Ciprofloxacin interacts with its gyrase/topoisomerase IV targets through a water-metal ion bridge.^19–21^ Each fluoroquinolone molecule chelates a divalent metal ion through its 3,4-ketoacid moiety. The divalent metal ion coordinates four water molecules, and two of these are coordinated by a conserved serine and acidic residue four amino acids apart in the GyrA/GrlA subunits of gyrase and topoisomerase IV, respectively.^19–22^ These residues (GyrA^S85^ and GyrA^E89^ in *B. anthracis* gyrase and GrlA^S81^ and GrlA^E85^ in *B. anthracis* topoisomerase IV) serve as the anchors for the water-metal ion bridge.^20,22^ They position the fluoroquinolone in the DNA cleavage/ligation active site of the type II enzymes in the vicinity of the scissile bonds. Because the type II topoisomerases must generate double-stranded DNA breaks to carry out their catalytic functions, the A subunits of gyrase (GyrA) and topoisomerase IV (GrlA) each contain two active site tyrosine residues.^16,23^ Once DNA scission occurs, separate drug molecules (one per DNA strand) are inserted between the 3′- and 5′- ends of the cleaved DNA.^16,23^

The insertion of ciprofloxacin and other fluoroquinolones into the cleaved scissile DNA bonds has two effects on the activities of gyrase and topoisomerase IV.^12,13,15^ First, the drug acts as a physical barrier to DNA ligation. As a result, the lifetime of the cleavage complex is extended from seconds (or subseconds) to minutes or even hours.^13,15,24^ This action has the potential to stabilize double-stranded DNA breaks throughout the bacterial chromosome, inducing the DNA damage SOS response.^12,13,25,26^ Second, because the drug inhibits DNA ligation, it impedes the overall catalytic activities of gyrase and topoisomerase IV.^12,13,15^ This latter action robs the bacterial cell of the essential functions of these enzymes, leading to delays in replication or impaired cell division.^12,13,15,24^ Although the induction of enzyme-mediated DNA cleavage is generally regarded as the more toxic effect of fluoroquinolones on bacteria, either of these actions has the potential to induce cell death.^27^

In addition to their use for treating anthrax,^5^ fluoroquinolones are one of the most efficacious broad spectrum oral antibacterials ever developed and are used to treat a variety of infections worldwide.^13,14,28^ Unfortunately, the clinical utility of this drug class for many infections is being curtailed by the rise of target-mediated resistance driven by mutations in gyrase and topoisomerase IV.^12,13,15,24,29^ The amino acids at which mutations are most often observed in clinical isolates are the serine and acidic residues in the bacterial type II topoisomerases that anchor the water-metal ion bridge.^12,13,15,24,30–32^ Due to the limited opportunity to obtain clinical isolates of *B. anthracis*, little information is available regarding resistance mutations. However, anthrax spores containing fluoroquinolone resistance mutations in gyrase and topoisomerase IV are a significant biological threat.^33,34^ Such mutations have the potential to render one of the frontline treatments against anthrax ineffective. Therefore, there is a need to develop new classes of antibacterials that are efficacious against both wild-type and fluoroquinolone-resistant *B. anthracis* infections.

One such new class of antibacterials is the triazaacenaphthylenes. The most clinically advanced triazaacenaphthylene is gepotidacin (Figure 1), which was recently approved by the U.S. Food and Drug Administration (FDA) and the U.K. Medicines and Healthcare products Regulatory Agency for the treatment of uncomplicated urinary tract infections in adult and adolescent females.^35–37^ In addition, a phase III clinical trial that evaluated the use of gepotidacin for the treatment of uncomplicated urogenital gonorrhea was completed with positive outcomes,^38,39^ and the drug has been accepted for priority review by the FDA for this infection.^40^ Like the fluoroquinolones, gepotidacin targets gyrase and topoisomerase IV and acts at the DNA cleavage-ligation site of the enzymes.^41–43^ However, gepotidacin interacts with different amino acid residues in gyrase and topoisomerase IV and stabilizes cleavage complexes by an alternative mechanism than fluoroquinolones.^41–43^

Gepotidacin was shown to be effective in a rabbit inhalation model of anthrax,^34^ suggesting that it is active against *B. anthracis* gyrase and topoisomerase IV. To determine whether compounds related to gepotidacin also display activity against anthrax, we examined the activity of OSUAB-0284 (Figure 1), a member of the Novel Bacterial Topoisomerase Inhibitor (NBTI) class and an emerging preclinical candidate that shares a general pharmacophore with gepotidacin.^44^ Recently, OSUAB-0284 was shown to display potent in vitro and in vivo activity against *Staphylococcus aureus* and other clinically relevant pathogens.^44^ Therefore, we expanded our studies on OSUAB-0284 to include *B. anthracis*. The NBTI displayed a potency against wild-type (WT) and fluoroquinolone-resistant *B. anthracis* cultures that was similar or greater than gepotidacin and was also efficacious in a mouse model of inhalation anthrax. OSUAB-0284 maintained activity against fluoroquinolone-resistant *B. anthracis* and was more potent than ciprofloxacin against purified recombinant gyrase and topoisomerase IV. The NBTI also induced high levels of enzyme-mediated DNA cleavage and maintained activity against *B. anthracis* gyrase and topoisomerase IV that harbored common fluoroquinolone-resistance mutations. Similar trends were observed with gepotidacin, which was used as a comparator for all enzymatic assays. Results suggest that OSUAB-0284, along with gepotidacin, may be potential candidates for the treatment of anthrax and provide mechanistic underpinnings supporting the use of both agents against this biothreat pathogen.

## Results and Discussion

### In Vitro Antibacterial Activity

As a first step toward assessing the potential use of OSUAB-0284 against anthrax, we assessed its in vitro antibacterial activity by determining minimal inhibitory concentrations (MICs) against a 30-strain panel of *B. anthracis* (Table 1, top). The related triazaacenaphthylene gepotidacin and the fluoroquinolone ciprofloxacin were included as comparators. Concentrations resulting in growth inhibition for 50% (MIC_50_) and 90% (MIC_90_) of the strains were calculated. Quality control (QC) strains were assessed in parallel with results in general agreement with available published ranges.

**Table 1:** Activity of Compounds against a Panel of 30 *B. anthracis* Strains (Top) and Susceptibility of a Panel of Fluoroquinolone-Resistant *B. anthracis* Strains to OSUAB-0284, Gepotidacin, and Ciprofloxacin (Bottom)

Drug	Range (μg/mL)	^a^MIC_50_ (μg/mL)	^a^MIC_90_ (μg/mL)
OSUAB-0284	≤0.03–4	0.06	0.25
Gepotidacin	0.12–8	0.5	0.5
Ciprofloxacin	0.015–0.25	0.03	0.06

	MIC (μg/mL)		
FQ-Resistant Strain	OSUAB-0284	Gepotidacin	Ciprofloxacin
Sterne	0.06	0.25	0.25
UT308	0.06	0.25	0.25
BACI389	0.5	8	8
BACI391	0.5	2	2
BACI392	1	8	>8
BACI393	0.125	1	>8
BACI396	0.125	0.5	>8
BACI420	0.25	2	4
BACI422	4	8	>8
BACI423	0.25	2	8
BACI424	0.25	2	8
BACI429	0.5	8	>8
BACI430	0.25	0.5	8
BACI432	0.25	1	2
BACI434	0.5	1	2
BACI436	4	32	>8
MIC	0.25	2	8
MIC	4	8	>8
Range^b^	0.06–4	0.25–32	2 to >8
%Susceptible	100%^c^	64%^c^	0%^d^

a Minimum inhibitory concentration (MIC) values of OSUAB-0284, gepotidacin and ciprofloxacin are shown for a 30-strain panel of *B. anthracis* cells. The observed range of MIC values for each compound over the entire panel are shown. MIC_50_ represents the concentration at which the growth of 50% of the strains in the panel were inhibited by 50%. MIC_90_ represents the concentration at which the growth of 90% of the strains in the panel were inhibited by 50%.

b Susceptibility percentages and range do not include parent strains Sterne or UT308.

c Using CLSI M45 *B. anthracis* ciprofloxacin breakpoint of ≤0.25 μg/mL.

d Using the FDA gepotidacin *E. faecalis*breakpoint of ≤4 μg/mL.

In most cases, OSUAB-0284 (MIC_50_ = 0.06 μg/mL) was more potent than gepotidacin (MIC_50_ = 0.5 μg/mL). However, MIC_90_ values of OSUAB-0284 and gepotidacin were similar (0.25 μg/mL vs 0.5 μg/mL respectively). Results with gepotidacin and ciprofloxacin (MIC_50_ = 0.03 μg/mL, MIC_90_ = 0.06 μg/mL) were consistent with previous studies.^34,45–47^

Antibacterial susceptibility was also assessed against Federal Select Agent Program (FSAP)-excluded, BSL-2 strains of *B. anthracis* that exhibit resistance to fluoroquinolones (Table 1, bottom). This BSL-2 panel was curated from a USAMRIID collection of surrogates with antimicrobial resistance (AMR) phenotypes that were generated from previous efforts using direct mutagenesis or serial passage with increasing concentrations of antibacterial agents. Full genetic characterization of this panel is pending and will be reported elsewhere. However, all strains (with the exception of the two parent strains) were resistant to ciprofloxacin according to CLSI published breakpoints.^48^

Both parent strains (UT308 and Sterne) were susceptible to ciprofloxacin (MIC = 0.25 μg/mL). In comparison, 64% and 100% of the strains were susceptible to gepotidacin and OSUAB-0284, respectively, when using the gepotidacin *E. faecalis* breakpoint as a surrogate for interpretation. The MIC value with OSUAB-0284 for any individual strain was between 2 and 16 times lower than those observed for gepotidacin, a difference similar to that observed in the virulent panel (Table 1). These results, together, suggest that OSUAB-0284 may have potential to treat anthrax including cases caused by fluoroquinolone-resistant strains of *B. anthracis*.

### Activity of OSUAB-0284, Gepotidacin, and Ciprofloxacin against Purified Wild-Type *B. anthracis* Gyrase

Triazaacenaphthylenes and NBTIs interact with gyrase and topoisomerase IV through amino acid residues that are not involved in the fluoroquinolone water-metal ion bridge.^23,41,49,50^ Unlike the fluoroquinolones, only a single triazaacenaphthylene or NBTI molecule binds to the type II enzymes. The left-hand side (Figure 1, LHS) of these compounds occupies a pocket in the DNA located on the 2-fold axis of the cleavage complex, midway between the two DNA cleavage active sites. The right-hand side (Figure 1, RHS) of these compounds represents the enzyme-binding motif and is positioned in the interface between the two GyrA/GrlA subunits. A critical interaction occurs between the basic nitrogen of gepotidacin and related compounds and an aspartic acid residue, which should be GyrA^D84^ and GrlA^D79^ in *B. anthracis* gyrase and topoisomerase IV, respectively.^23,41,51–53^ As a result of this interaction, gepotidacin and NBTIs stabilize primarily single-stranded DNA breaks by gyrase and topoisomerase IV.^41,42,51,53^ Although their mechanism of action has not been definitively established, it is believed that these compounds distort the cleavage complex in a manner that inhibits the ability of gyrase and topoisomerase IV to ligate the nicked DNA.^41^ This distortion also inhibits the overall catalytic activities of the type II enzymes.^41–43^

Because of the antibacterial activity of OSUAB-0284 and gepotidacin against *B. anthracis* in vitro, we determined their activities against gyrase and topoisomerase IV in parallel to ciprofloxacin. The first experiments determined the effects of these compounds on DNA cleavage mediated by gyrase (Figure 2).

**Figure 2:**
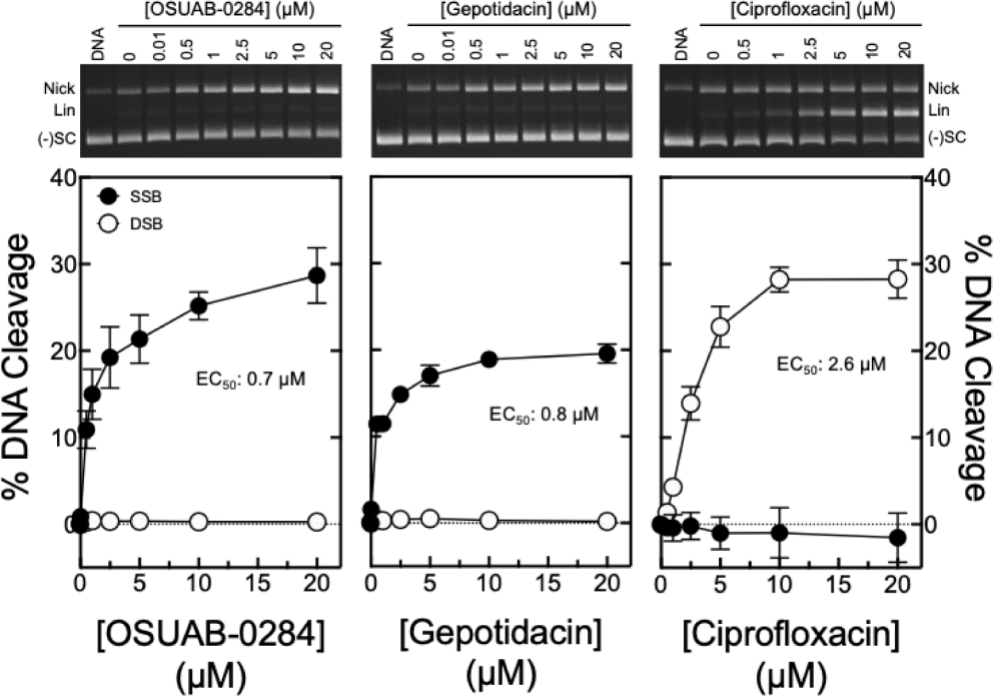
Effects of OSUAB-0284, gepotidacin, and ciprofloxacin on DNA cleavage mediated by wild-type *B. anthracis* gyrase. Levels of single-stranded (filled circles, SSB) and double-stranded DNA breaks (open circles, DSB) induced by OSUAB-0284 (left panel), gepotidacin (middle panel), and ciprofloxacin (right panel) are shown. Error bars represent the standard deviation of at least 3 independent experiments. A representative gel of gyrase-mediated DNA cleavage with each compound is shown above the corresponding graph. DNA represents the negatively supercoiled DNA control. The positions of nicked (Nick), linear (Lin), and negatively supercoiled [(−)­SC] plasmid are indicated. Levels of DNA cleavage were adjusted for baseline cleavage in the absence of drugs.

OSUAB-0284 (left panel) and gepotidacin (middle panel) both induced primarily single-stranded DNA breaks mediated by *B. anthracis* gyrase. This is as opposed to ciprofloxacin (right panel), which induced primarily double-stranded DNA breaks. As determined by their EC_50_ values (effective concentration that induces 50% maximal DNA cleavage) OSUAB-0284 (EC_50_ ≈ 0.7 μM) and gepotidacin (EC_50_ ≈ 0.8 μM) were 2–3-fold more potent than ciprofloxacin (EC_50_ ≈ 2.6 μM). OSUAB-0284 induced more single-stranded DNA breaks compared to gepotidacin (∼30% vs ∼20% maximal cleavage adjusted for baseline DNA cleavage levels generated in the absence of drug). These levels were comparable to the adjusted maximal double-stranded DNA cleavage induced by ciprofloxacin (∼30%).

The inhibition of gyrase catalytic activity by antibacterial agents can also affect drug efficacy.^27^ Thus, we examined the ability of the three compounds to inhibit DNA supercoiling catalyzed by *B. anthracis* gyrase (Figure 3). Once again, OSUAB-0284 (left panel; IC_50_ ≈ 6.3 μM) was more potent than gepotidacin (middle panel; IC_50_ ≈ 23.2 μM). However, both compounds were considerably more potent inhibitors than ciprofloxacin (right panel; IC_50_ ≈ 118 μM).

**Figure 3:**
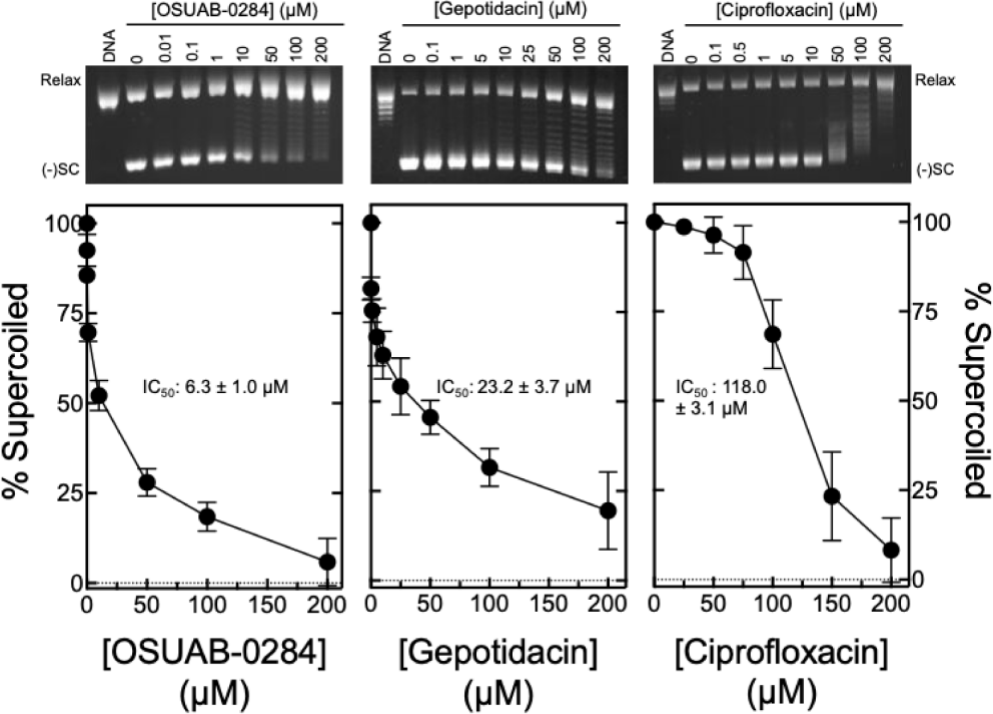
Effects of OSUAB-0284, gepotidacin, and ciprofloxacin on DNA supercoiling catalyzed by wild-type *B. anthracis* gyrase. The inhibition of catalytic activity by OSUAB-0284 (left panel), gepotidacin (middle panel), and ciprofloxacin (right panel) is shown. Error bars represent the standard deviation of at least 3 independent experiments. IC_50_ values (drug concentration at which enzyme activity is inhibited by 50%), including the standard error of the mean, for each assay are shown in the respective panels. A representative gel of gyrase-catalyzed DNA supercoiling with each compound is shown above the corresponding graph. DNA represents the fully relaxed DNA control. The positions of relaxed (Relax) and negatively supercoiled [(−)­SC] plasmids are indicated.

### Activity of OSUAB-0284, Gepotidacin, and Ciprofloxacin against Purified Wild-Type *B. anthracis* Topoisomerase IV

Although gyrase is the primary cellular target for fluoroquinolones in *B. anthracis*, topoisomerase IV appears to be a secondary target.^54–56^ This “unbalanced” targeting allows stepwise mutations in the bacterial type II topoisomerases that result in the evolution of highly resistant *B. anthracis* strains.^54–56^ Although gepotidacin displays balanced dual-targeting in *Escherichia coli*, *Klebsiella pneumoniae* and *Neisseria gonorrhoeae* (i.e., the drug can kill bacterial cells through its effects on either gyrase or topoisomerase IV),^42,43,57,58^ the targeting of gepotidacin and OSUAB-0284 in *B. anthracis* has not been described. Therefore, the abilities of these two compounds to induce DNA cleavage mediated by *B. anthracis* topoisomerase IV were assessed (Figure 4).

**Figure 4:**
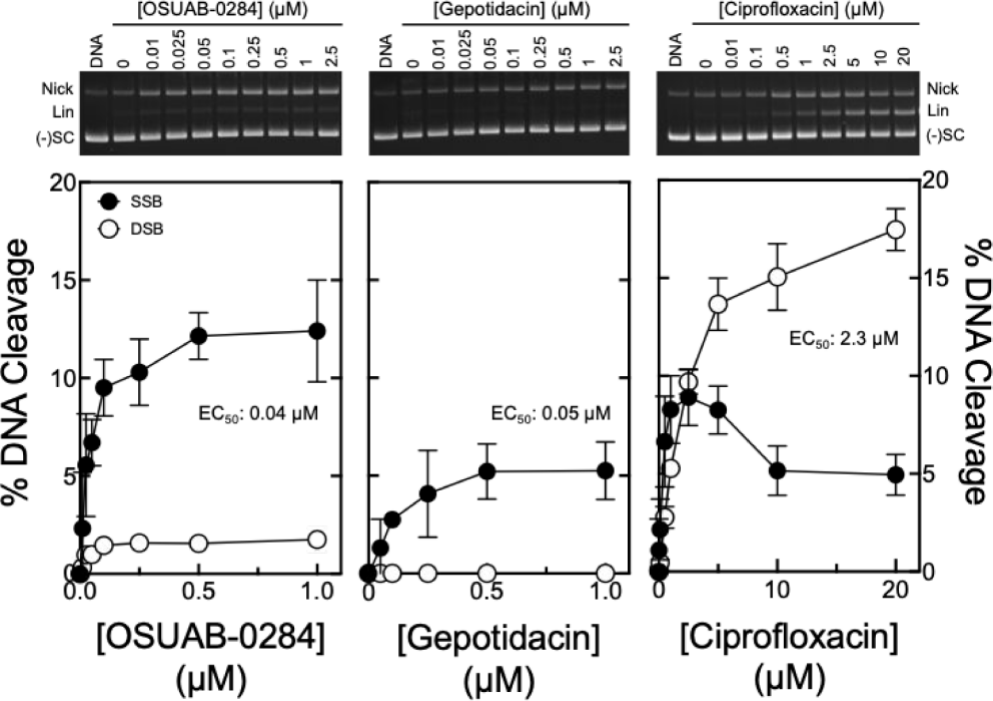
Effects of OSUAB-0284, gepotidacin, and ciprofloxacin on DNA cleavage mediated by wild-type *B. anthracis* topoisomerase IV. Levels of single-stranded (filled circles, SSB) and double-stranded DNA breaks (open circles, DSB) induced by OSUAB-0284 (left panel), gepotidacin (middle panel), and ciprofloxacin (right panel) are shown. Error bars represent the standard deviation of at least 3 independent experiments. A representative gel of topoisomerase IV-mediated DNA cleavage with each compound is shown above the corresponding graph. DNA represents the negatively supercoiled DNA control. The positions of nicked (Nick), linear (Lin), and negatively supercoiled [(−)­SC] plasmid are indicated. Levels of DNA cleavage were adjusted for baseline cleavage in the absence of drugs.

OSUAB-0284 (left panel; EC_50_ ≈ 0.04 μM) and gepotidacin (middle panel; EC_50_ ≈ 0.05 μM) displayed similar potencies against topoisomerase IV. Furthermore, both were >50-fold more potent than ciprofloxacin (right panel; EC_50_ ≈ 2.3 μM). Once again, OSUAB-0284 and gepotidacin induced primarily single-stranded DNA breaks. As seen with gyrase, OSUAB-0284 (∼12% maximal cleavage adjusted for baseline DNA cleavage levels generated in the absence of drug) generated higher levels of single-stranded DNA breaks compared to gepotidacin (∼5% adjusted maximal DNA cleavage). Ciprofloxacin induced primarily double-stranded DNA breaks at levels that were higher (∼17% adjusted maximal cleavage).

It is notable that in addition to single-stranded DNA breaks, OSUAB-0284 induced low levels (∼2%) of double-stranded breaks. The induction of double-stranded DNA breaks has been observed previously for some other NBTIs.^51,53,59,60^

OSUAB-0284 (IC_50_ ≈ 0.1 μM) and gepotidacin (IC_50_ ≈ 0.2 μM) displayed similar potencies as catalytic inhibitors of DNA decatenation catalyzed by *B. anthracis* topoisomerase IV (Figure 5). As seen for DNA cleavage, both the NBTI and triazaacenaphthylene were considerably more potent than ciprofloxacin (IC_50_ ≈ 178 μM).

**Figure 5:**
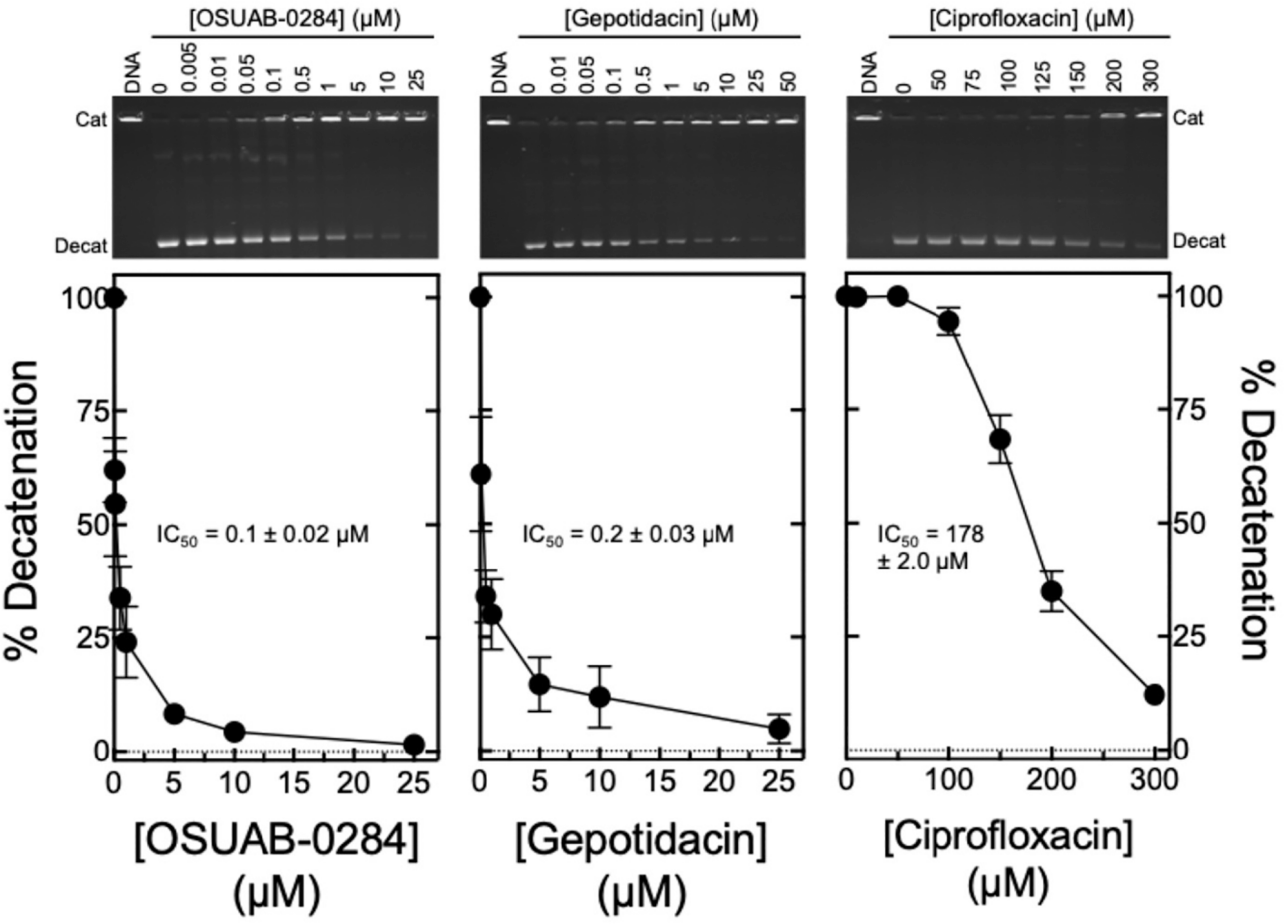
Effects of OSUAB-0284, gepotidacin, and ciprofloxacin on DNA decatenation catalyzed by wild-type *B. anthracis* topoisomerase IV. The inhibition of catalytic activity by OSUAB-0284 (left panel), gepotidacin (middle panel), and ciprofloxacin (right panel) is shown. Error bars represent the standard deviation of at least 3 independent experiments. IC_50_ values (drug concentration at which enzyme activity is inhibited by 50%), including the standard error of the mean, for each assay are shown in the respective panels. A representative gel of topoisomerase IV-catalyzed decatenation with each compound is shown above the corresponding graph. DNA represents the fully catenated (Cat) DNA control. The position of decatenated (Decat) DNA is indicated.

Finally, OSUAB-0284 and gepotidacin were substantially more potent against topoisomerase IV than gyrase. Although it will require genetic studies to definitively address the issue of cellular targeting, this large disparity in potency against the two enzymes suggests that the NBTI and triazaacenaphthylene might not display balanced dual-targeting in this species.

### Activity of OSUAB-0284 and Gepotidacin against Purified *B. anthracis* Gyrase and Topoisomerase IV Enzymes that Carry Fluoroquinolone-Resistance Mutations

Previous studies with cells and purified enzymes demonstrated that mutations in the serine (GyrA^S85L^, GrlA^S81Y^) and glutamic acid (GyrA^E89K^, GrlA^E85K^) residues that anchor the water-metal ion bridge in *B. anthracis* gyrase and topoisomerase IV, respectively, diminish the activities of fluoroquinolones.^21,22,61^ The mutations significantly reduced the potency of these drugs against gyrase and topoisomerase IV and their ability to induce enzyme-mediated DNA cleavage.^22,61^ Because of concerns that *B. anthracis* strains carrying fluoroquinolone-resistance mutations could be misused, the activities of OSUAB-0284 and gepotidacin against fluoroquinolone-resistant gyrase and topoisomerase IV were determined.

*Gyrase—*OSUAB-0284 induced higher levels of single-stranded DNA cleavage mediated by GyrA^S85L^ (∼31% maximal cleavage adjusted for baseline DNA cleavage levels generated in the absence of drug) than wild-type gyrase (∼25% adjusted maximal DNA cleavage) and was nearly 2-fold more potent (EC_50_ ≈ 0.3 μM vs 0.7 μM) (Figure 6A, left panel). The NBTI maintained activity against GyrA^E89K^, albeit with a slight decrease in efficacy (∼21% adjusted maximal DNA cleavage) and a ∼5-fold reduction in potency (EC_50_ ≈ 1.6 μM). Gepotidacin maintained activity against GyrA^S85L^, displaying similar adjusted maximal levels of single-stranded DNA cleavage (∼18%) with considerably greater potency (EC_50_ ≈ 0.1 μM) than observed with the wild-type enzyme (EC_50_ ≈ 0.8 μM). As seen with OSUAB-0284, gepotidacin induced lower levels of DNA cleavage with GyrA^E89K^ (Figure 6A, right panel) but maintained its potency (EC_50_ ≈ 0.7 μM).

**Figure 6:**
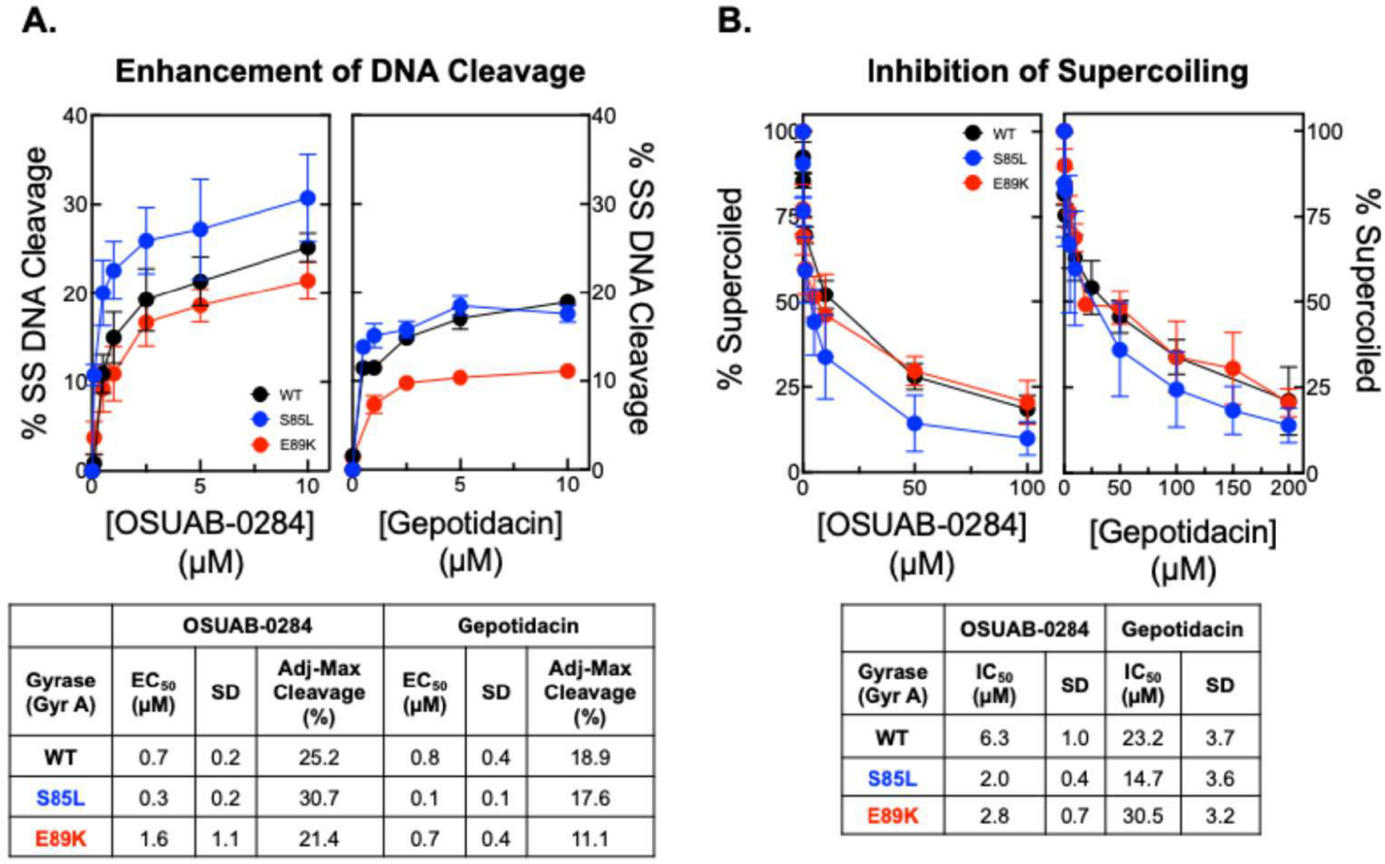
Effects of OSUAB-0284, gepotidacin, and ciprofloxacin on the activities of WT and fluoroquinolone-resistant mutants of *B. anthracis* gyrase. (A) Levels of single-stranded DNA breaks induced by OSUAB-0284 (left panel) and gepotidacin (right panel) are shown for WT gyrase (black) and fluoroquinolone-resistant mutant enzymes that harbor GyrA^S85L^ (blue) or GyrA^E89K^ (red). A summary table displays the EC_50_ and adjusted maximal DNA cleavage values. (B) The inhibition of supercoiling by OSUAB-0284 (left panel) and gepotidacin (right panel) are shown for wild-type gyrase (black) and fluoroquinolone-resistant mutant enzymes that harbor GyrA^S85L^ (blue) or GyrA^E89K^ (red). Error bars represent the standard deviation of at least 3 independent experiments. A summary table displays IC_50_ values (drug concentration at which enzyme activity is inhibited by 50%), including the standard error of the mean, for each assay.

Both OSUAB-0284 and gepotidacin maintained activity in catalytic inhibition experiments with gyrase (Figure 6B). OSUAB-0284 (Figure 6B, left panel) displayed greater potency against both the GyrA^S85L^ (IC_50_ ≈ 2.0 μM) and GyrA^E89K^ (IC_50_ ≈ 2.8 μM) mutant enzymes compared to wild-type gyrase (IC_50_ ≈ 6.3 μM). Gepotidacin (Figure 6B, right panel) displayed greater potency against the GyrA^S85L^ (IC_50_ ≈ 14.7 μM) mutant enzyme compared to wild-type gyrase (IC_50_ ≈ 23.2 μM), with only a slight decrease in potency against GyrA^E89K^ (IC_50_ ≈ 30.5 μM).

*Topoisomerase IV—*Both OSUAB-0284 (Figure 7A, left panel) and gepotidacin (Figure 7A, right panel) induced higher levels of DNA cleavage with fluoroquinolone-resistant topoisomerase IV mutant enzymes (GrlA^S81Y^, GrlA^E85K^) compared to wild-type topoisomerase IV. OSUAB-0284, induced levels of single-stranded DNA breaks with GrlA^S81Y^ (∼24% maximal cleavage adjusted for baseline DNA cleavage levels generated in the absence of drug, EC_50_ ≈ 0.06 μM) and GrlA^E85K^ (∼19% adjusted maximal DNA cleavage, EC_50_ ≈ 0.01 μM) that were ∼1.5–2-fold higher than observed with wild-type topoisomerase IV (∼12% adjusted maximal DNA cleavage, EC_50_ ≈ 0.04 μM) with similar or greater potencies. Gepotidacin also induced higher levels of single-stranded DNA cleavage by GrlA^S81Y^ (∼12% adjusted maximal DNA cleavage, EC_50_ ≈ 0.05 μM) and GrlA^E85K^ (∼12% adjusted maximal DNA cleavage, EC_50_ ≈ 0.05 μM) compared to wild-type topoisomerase IV (∼5% adjusted maximal DNA cleavage, EC_50_ ≈ 0.1 μM) and maintained its potency against the mutant enzymes.

**Figure 7:**
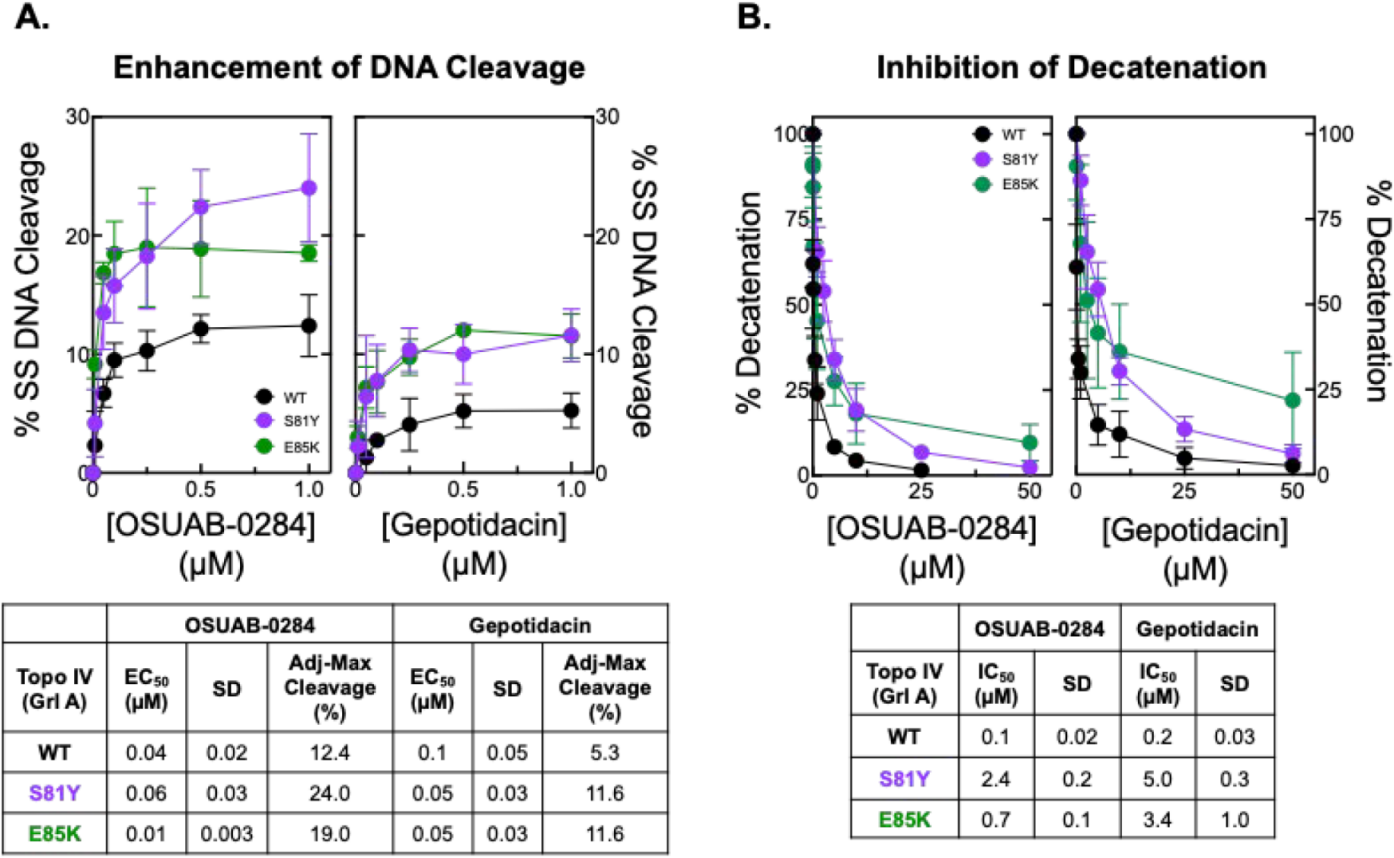
Effects of OSUAB-0284, gepotidacin, and ciprofloxacin on the activities of WT and fluoroquinolone-resistant mutants of *B. anthracis* topoisomerase IV. (A) Levels of single-stranded DNA breaks induced by OSUAB-0284 (left panel) and gepotidacin (right panel) are shown for WT gyrase (black) and fluoroquinolone-resistant mutant enzymes that harbor GyrA^S81Y^ (purple) or GyrA^E85K^ (green). Error bars represent the standard deviation of at least 3 independent experiments. A summary table displays the EC_50_ and adjusted maximal DNA cleavage values. (B) The inhibition of decatenation by OSUAB-0284 (left panel) and gepotidacin (right panel) are shown for WT topoisomerase IV (black) and fluoroquinolone-resistant mutant enzymes that harbor GyrA^S81Y^ (purple) and GyrA^E85K^ (green). Error bars represent the standard deviation of at least 3 independent experiments. A summary table displays IC_50_ values (drug concentration at which enzyme activity is inhibited by 50%), including the standard error of the mean, for each assay.

In contrast to DNA cleavage, both OSUAB-0284 and gepotidacin were less potent inhibitors of the mutant topoisomerase IV enzymes (Figure 7B, left and right panels, respectively). IC_50_ values for OSUAB-0284 rose several-fold with GrlA^S81Y^ (IC_50_ ≈ 2.4 μM), and GrlA^E85K^ (IC_50_ ≈ 0.7 μM) compared to wild-type (IC_50_ ≈ 0.1 μM). Similar trends were observed with gepotidacin (GrlA^S81Y^ IC_50_ ≈ 5.0 μM; GrlA^E85K^ IC_50_ ≈ 3.4 μM; WT IC_50_ ≈ 0.2 μM). The largest effects were seen with the GrlA^S81Y^ mutant. However, even with these prototypical fluoroquinolone-resistant mutants, both compounds were still relatively potent with IC_50_ values not exceeding the single digit micromolar range.

Collectively, the above data provide strong evidence that OSUAB-0284 and gepotidacin maintain their activity against *B. anthracis* gyrase and topoisomerase IV that harbor the most common fluoroquinolone-resistance mutations in amino acid residues that anchor the water-metal ion bridge.

### In Vivo Pharmacology of OSUAB-0284 in BALB/c Mice

As the next step toward assessing the potential for OSUAB-0284 to be used as a treatment for anthrax, a mouse anthrax inhalation model was utilized. To inform subsequent dosing strategies in our infection model, a pharmacokinetic study was carried out in BALB/c mice. Mice were administered single doses of OSUAB-0284 either by intravenous (IV) tail-vein injection (10 mg/kg) or oral gavage (10 or 50 mg/kg). Plasma concentrations of OSUAB-0284 were quantified using Liquid Chromatography-Tandem Mass Spectrometry (LC-MS/MS). Results are shown in Figure 8.

**Figure 8:**
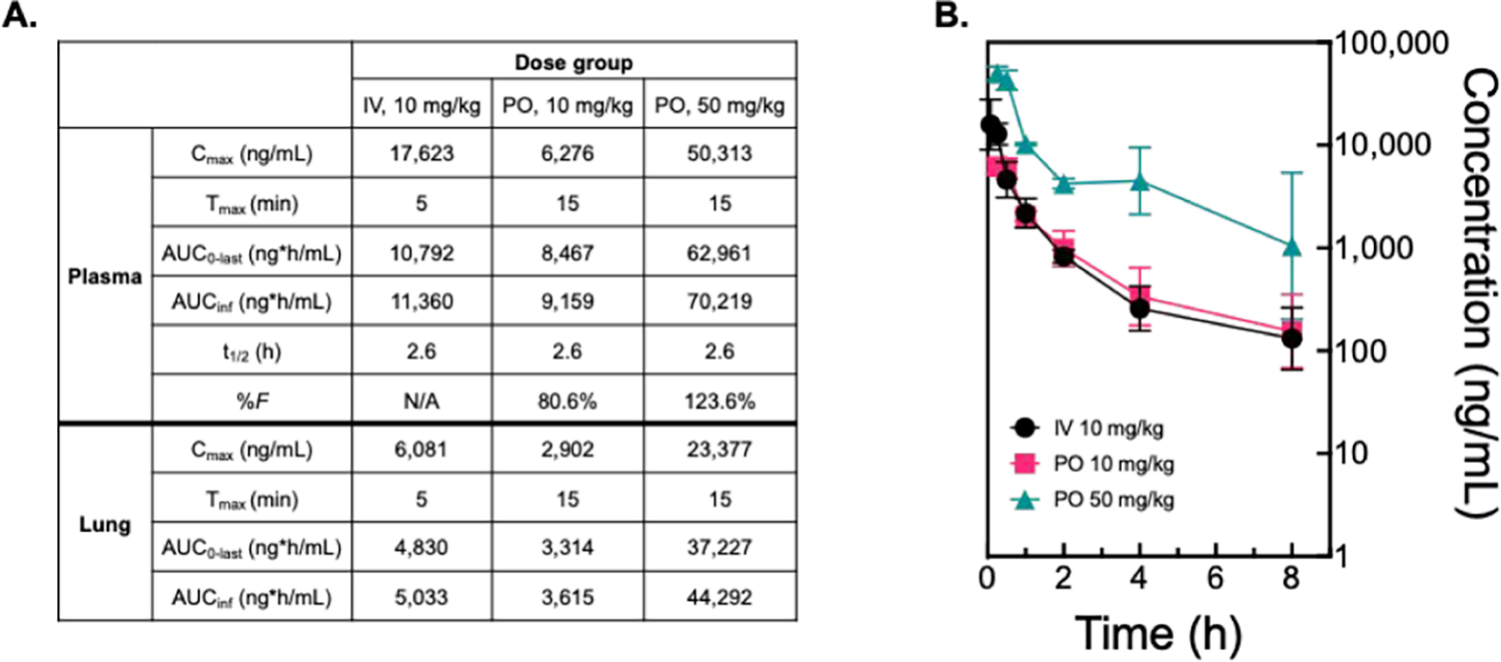
In vivo pharmacokinetics of OSUAB-0284. (A) Table of in vivo pharmacokinetic parameters of OSUAB-0284 in BALB/c mice following single IV (10 mg/kg) or oral doses (10 mg/kg or 50 mg/kg). (B) Concentration versus time curves of OSUAB-0284 plasma concentrations following single IV (10 mg/kg, black) or oral doses (10 mg/kg, pink or 50 mg/kg, teal) BALB/c mice.

OSUAB-0284 demonstrated high oral bioavailability (*F* = 80.6%) as determined by the area under the curve (AUC_0‑inf_) for the matched 10 mg/kg oral versus intravenous doses. Between the 50 mg/kg and 10 mg/kg oral groups, the pharmacokinetics displayed nonproportionality with the dose-corrected exposure 1.5-fold greater in the higher vs lower dose group. This nonproportionality in the 50 mg/kg group may be linked to higher variability in the terminal elimination phase. In all groups, significant exposure was also observed in lung homogenates, suggesting the potential use of OSUAB-0284 to treat pulmonary infections (AUC_0‑∞_^Lung^/AUC_0‑∞_^Plasma^ = 36–53%). Plasma protein binding was determined to be 69% and the half-life (*t*_1/2_) of OSUAB-0284 was 2.6 h.^62^

The pharmacokinetics of OSUAB-0284 supported twice daily oral administration (BID) in mice. A pilot study in mice was initiated to assess drug tolerability with BID oral dosing (Figure 8B). No adverse events or cage-side observations were noted in cohorts of BALB/c mice receiving up to the highest tested dose of 100 mg/kg (PO, BID) for 3 days. Two oral doses were selected for efficacy testing in mice. A high dose of 100 mg/kg (BID, PO) was selected as it was the highest tested dose in the pilot safety study for which no adverse events were noted. A human-equivalent dose of 34.7 mg/kg (PO, BID) of OSUAB-0284 was calculated to match the estimated free exposure of twice daily 1500 mg oral gepotidacin.^63,64^

### In Vivo Activity of OSUAB-0284 in an Inhalation Anthrax Mouse Model

In a previous study, gepotidacin was highly efficacious in a rabbit model of inhalation anthrax.^34^ Ten out of 11 rabbits treated with gepotidacin survived for at least 30 days, whereas control animals succumbed or were euthanized in accordance with early intervention criteria within 5 days post challenge. The lone gepotidacin treated animal that died (3 days postchallenge) suffered complications with catheter administration that negatively impacted infusion start time and drug exposure. Given that OSUAB-0284 displayed similar or greater activity than gepotidacin against cultured *B. anthracis* and purified wild-type and fluoroquinolone-resistant *B. anthracis* gyrase and topoisomerase IV, we utilized a BALB/c mouse model to determine the efficacy of OSUAB-0284 against inhalation anthrax.

The BALB/c model remains the gold-standard small-animal in vivo model for initial evaluation of therapeutics against inhalational anthrax and was used here to assess efficacy of OSUAB-0284. Forty BALB/c mice were challenged with *B. anthracis* (Ames strain) via whole-body aerosol at an average inhaled dose of 103.6 × LD_50_ (50% lethal dose). Treatments were initiated 24 h postchallenge and included groups of 10 mice that were treated intraperitoneally (IP) with either saline or standard-of-care ciprofloxacin (30 mg/kg, BID) as negative and positive controls, respectively. OSUAB-0284 cohorts were treated orally (PO) with either 34.7 or 100 mg/kg (BID). All groups were treated for a total of 14 days. Survival was assessed twice daily during the treatment period of 14 days and at least once daily during the observation period from day 15 to day 30.

All saline-treated mice succumbed by 48 h postchallenge (Figure 9A). In contrast, all 10 of the ciprofloxacin-treated mice survived study termination on day 30. Both OSUAB-0284 regimens were highly protective against inhalation anthrax with 100% and 90% survival in the 34.7 and 100 mg/kg groups, respectively. Survival in neither the high (*P* = 1.0) nor low (*P* = 0.32) groups were statistically different than ciprofloxacin.

**Figure 9:**
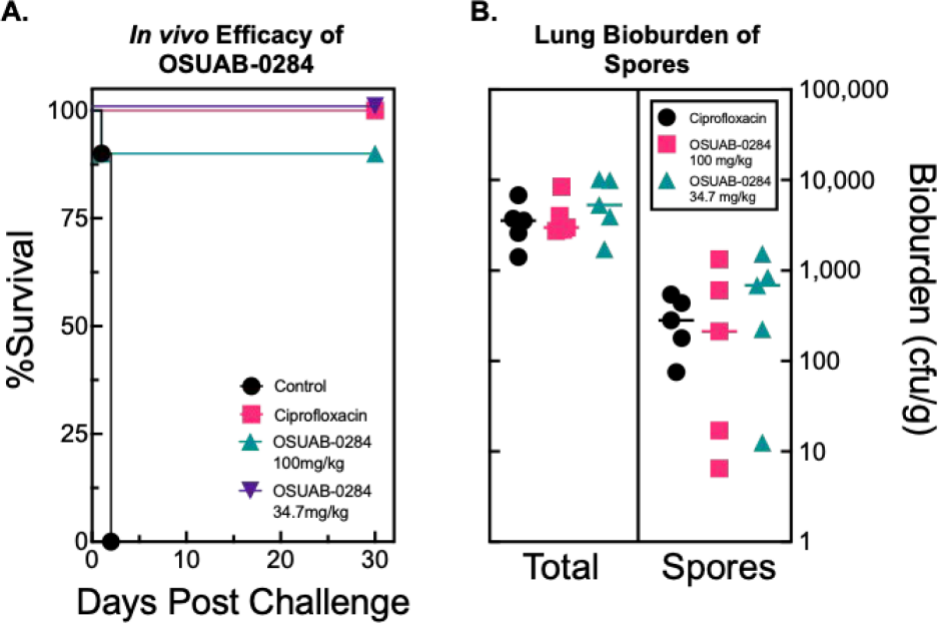
In vivo efficacy of OSUAB-0284 and ciprofloxacin. (A) Aerosol challenge of *B. anthracis* spores in BALB/c mice treated with saline control (black), ciprofloxacin (30 mg/kg, pink), OSUAB-0284 (100 mg/kg, teal), OSUAB-0284 (34.7 mg/kg, purple). (B) Lung bioburden of spores and total *B. anthracis* (spores and vegetative cells) in surviving in BALB/c mice by cohort. Dose levels of OSUAB-0284 are as indicated in Panel (A).

At study termination, the spore and total *B. anthracis* bioburden was assessed in five mice per surviving group. Measurable levels of vegetative cells and spores were found in all lung tissue samples examined (Figure 9B). No significant differences in total bacterial burden (*P* = 0.28–0.93) or spore counts (*P* = 0.42–0.88) between the ciprofloxacin and OSUAB-0284 groups were observed. This finding is consistent with reported delayed spore germination and spore persistence in animals and humans.^9,65^ Spore persistence is the reason longer treatment times (up to 60 days) are recommended for postexposure prophylaxis for inhalation anthrax. Furthermore, no mice showed any clinical signs of disease at the termination of the study. The high level of protection afforded by orally administered OSUAB-0284 against a uniformly lethal challenge of aerosolized anthrax, combined with in vitro activity against ciprofloxacin-resistant strains, makes OSUAB-0284 an attractive candidate for further development as a medical countermeasure against anthrax infections.

## Conclusions

*B. anthracis* is the causative agent of anthrax and remains a biological threat.^1–3^ The fluoroquinolone ciprofloxacin is currently the prophylactic drug of choice against potential anthrax exposure.^5^ However, anthrax spores harboring fluoroquinolone-resistant gyrase and topoisomerase IV pose a significant threat to both public and military health and there is a critical need to identify new classes of antibacterials that function against this pathogen. Recently, the triazaacenaphthylene gepotidacin was shown to be highly active in vitro against *B. anthracis* vegetative cells and in a rabbit inhalation anthrax model.^34^ The work described in the current study elucidates the mechanistic basis for the activity of gepotidacin against wild-type and fluoroquinolone-resistant mutants of *B. anthracis* gyrase and topoisomerase IV.

To further explore the use of nonfluoroquinolone antibacterials for the treatment of anthrax, the current study extends the work on gepotidacin to the related compound OSUAB-0284. This NBTI displayed potent MIC values against wild-type and fluoroquinolone-resistant *B. anthracis* bacteria and activity against purified gyrase, and topoisomerase IV that was comparable to or greater than that of gepotidacin. Furthermore, in a mouse model of inhalation anthrax, orally administered OSUAB-0284 afforded significant protection (90–100% survival) that was statistically comparable to the standard-of-care ciprofloxacin (intraperitoneal administered). Taken together, our data further validate the potential use of gepotidacin against *B. anthracis* infections and strongly suggest that OSUAB-0284 offers another possible novel oral treatment option against anthrax.

## Materials and Methods

### DNA, Materials, and Enzymes

Negatively supercoiled pBR322 DNA was prepared from *E. coli* using a Plasmid Mega Kit (Qiagen) as described by the manufacturer. Relaxed pBR322 plasmid was generated by treating negatively supercoiled pBR322 with calf thymus topoisomerase I (Invitrogen) in 10 mM Tris-HCl (pH 7.9), 175 mM KCl, 5 mM MgCl_2_, 2.5% glycerol for 60 min at 37 °C followed by heat inactivation of topoisomerase I at 75 °C for 15 min.^66^ Kinetoplast DNA (kDNA) was isolated from *Crithidia fasciculata* as described by Englund.^67^

Ciprofloxacin (Sigma) was stored at −20 °C as a 40 mM stock solution in 0.1 M NaOH and diluted 5-fold with 10 mM Tris-HCl (pH 7.9) immediately prior to use. Gepotidacin (MedChemExpress) was stored at −20 °C as a 6 mM stock solution in 100% DMSO. OSUAB-0284, 6-(((*trans*-2-((*S*)-2-(3-fluoro-6-methoxy-1,5-naphthyridin-4-yl)-1-hydroxyethyl)-1,3-dioxan-5-yl)­amino)­methyl)-2*H*-pyrazino­[2,3-*b*]­[1,4]­oxazin-3­(4*H*)-one, was synthesized using methods previously reported by West et al.^44^ The compound was stored at −20 °C as 12 mM stock solution in 100% DMSO. Gepotidacin and OSUAB-0284 were diluted in H_2_O to 50% DMSO and further diluted in 50% DMSO to achieve desired 10× drug concentrations prior to addition to assay mixtures. The final percentage of DMSO in assays was 5%. All other chemicals were analytical reagent grade.

All proteins were 6× His-tagged. *B. anthracis* wild-type gyrase (GyrA, GyrB) and topoisomerase IV (GrlA, GrlB) subunits as well as mutant GyrA^S85L^ and GyrA^E89K^ gyrase subunits, and GrlA^S81Y^, GrlA^E85K^ topoisomerase IV subunits (QuikChange II XL site-directed mutagenesis, Agilent Technologies) were prepared as described previously.^22,61^ The identities of all constructs were confirmed by DNA sequencing. All gyrase subunits were stored in buffer containing 50 mM Tris-HCl (pH 7.5), 200 mM NaCl, 20% glycerol, and 5 mM dithiothreitol (DTT) and kept at −80 °C.^22^ All topoisomerase IV subunits were stored in buffer containing 20 mM Tris-HCl (pH 7.5), 200 mM NaCl and 20% glycerol and stored at −80 °C.^61^ All enzyme stock solutions were prepared at 20× concentration to achieve 1× concentration in final reactions. The stated concentration of gyrase and topoisomerase IV in reaction mixtures reflects that of the holoenzyme (A_2_B_2_). All wild-type DNA gyrase supercoiling experiments utilized enzyme made by GenScript.

### Antimicrobial Susceptibility Testing

All bacterial strains used for antimicrobial susceptibility testing were held by the United States Army Medical Research Institute of Infectious Diseases (USAMRIID) Bacteriology Division. For testing in BSL-3, a 30-strain panel of *B. anthracis* was assembled to evaluate activity across geographically diverse isolates. This panel includes the Ames strain used for in vivo efficacy testing. The Ames strain was originally isolated from a dead cow in Texas and was acquired by USAMRIID from the U.S. Department of Agriculture.^68^ For testing in BSL-2, FSAP-exempt strains of *B. anthracis* with AMR phenotypes were assembled. All BSL-2 AMR *B. anthracis* strains were in either the Sterne or UT308 background. The BSL-2 panel was assembled from a previous collection, and strains were selected based on having attenuated susceptibility as defined as ≥4-fold increase in ciprofloxacin MICs.^69^ MICs were determined by the CLSI microbroth dilution method in cation-adjusted Mueller-Hinton Broth (CAMHB) as previously described.^46,48,70,71^ Briefly, strains were streaked for isolation from a frozen glycerol stock onto a sheep blood agar plate and incubated at 35 ± 2 °C for 18–24 h. Antibacterials were serially diluted 2-fold on a 96-well plate in CAMHB prior to inoculation with bacteria in media. Final concentrations of the antibacterials tested were: 64–0.03 μg/mL for OSUAB-0284 and gepotidacin; 8–0.004 μg/mL for ciprofloxacin (USP). 96-well plates were incubated at 35 ± 2 °C for 16–20 h. The endpoint for an MIC microdilution was visually determined by a negative growth well prior to a positive growth well for each test article. Standard QC strains were evaluated in parallel with all MIC tests to include *Escherichia coli* (ATCC 25922), *Staphylococcus aureus* (ATCC 29213), and *Pseudomonas aeruginosa* (ATCC 27853). Where available, resulting QC values were generally within published ranges.^46,48,70,71^ To aid interpretation and where appropriate, *E. faecalis* breakpoint was leveraged as a comparator for MIC values because it is considered to be more consistent with published pharmacokinetic-pharmacodynamic profiles from lung infection models such as community-acquired bacterial pneumonia (CABP). Furthermore, *E. faecalis* has an MIC distribution more similar to *B. anthracis* compared to other Gram-positive organisms for which gepotidacin has approved breakpoints.^72^

### DNA Cleavage

DNA cleavage reactions were performed according to previously published procedures.^22,61^ Reactions were performed in the absence or presence of increasing concentrations of OSUAB-0284, gepotidacin, or ciprofloxacin. Gyrase DNA cleavage assay mixtures contained 10 nM negatively supercoiled pBR322 and *B. anthracis* gyrase [500 nM wild-type GyrA, GyrA^S85L^, or GyrA^E89K^ and 1000 nM wild-type GyrB] in a total volume of 20 μL of 50 mM Tris-HCl (pH 7.5), 100 mM KGlu, 5 mM MgCl_2_, and 50 μg/mL bovine serum albumin (BSA). Topoisomerase IV DNA cleavage assay mixtures contained *B. anthracis* topoisomerase IV [150 nM wild-type GrlA, GrlA^S81Y^, or GrlA^E85K^ and 300 nM GrlB] in a total volume of 20 μL of 40 mM Tris-HCl (pH 7.9), 50 mM NaCl, 2.5% (w/v) glycerol, and 10 mM MgCl_2_.

Reactions were incubated at 37 °C for 30 min with wild-type and mutant (GyrA^S85L^ or GyrA^E89K^) *B. anthracis* gyrase and 10 min with wild-type and mutant (GrlA^S81Y^or GrlA^E85K^) *B. anthracis* topoisomerase IV. Enzyme-DNA cleavage complexes were trapped by adding 2 μL of 5% SDS followed by 2 μL of 250 mM EDTA (pH 8.0). Proteinase K was added (2 μL of a 0.8 mg/mL solution), and reaction mixtures were incubated at 45 °C for 30 min to digest the enzyme. Samples were mixed with 2 μL of loading buffer (60% sucrose, 10 mM Tris-HCl (pH 7.9), 0.5% bromophenol blue, and 0.5% xylene cyanol FF) and heated at 45 °C for 2 min prior to electrophoresis in 1% agarose gels in 40 mM Tris-acetate (pH 8.3) and 2 mM EDTA containing 0.5 μg/mL ethidium bromide. DNA bands were visualized by midrange ultraviolet light and quantified using an Alpha Innotech digital imaging system (Protein Simple). Double-stranded DNA cleavage was monitored by the conversion of negatively supercoiled to linear plasmid molecules. EC_50_ values (the concentration of drug that induced 50% maximal DNA cleavage) were calculated using GraphPad Prism Version 10.0.3 utilizing a nonlinear regression analysis with 95% confidence intervals.

### Gyrase-Catalyzed DNA Supercoiling

DNA supercoiling assays were based on previously published protocols by Aldred et al.^61^ Reactions were performed in the absence or presence of increasing concentrations of OSUAB-0284, gepotidacin, and ciprofloxacin. Assay mixtures contained 50 nM wild-type (GyrA:GyrB) (1:1) or 300 nM mutant (GyrA^S85L^:GyrB, GyrA^E89K^:GyrB) (1:2 ratio) *B. anthracis* gyrase, 5 nM relaxed pBR322, 5 mM DTT, and 1.5 mM ATP in a total volume of 20 μL of 50 mM Tris-HCl (pH 7.5), 175 mM KGlu, 5 mM MgCl_2_, and 50 μg/mL BSA. Assay mixtures were incubated at 37 °C for 30 min with wild-type enzyme, and 60 min with mutant *B. anthracis* (GyrA^S85L^ or GyrA^E89K^), which represents the minimum time required to supercoil at least 90% of the DNA in the absence of drug. Reactions were stopped by the addition of 3 μL of a mixture of 0.77% SDS and 77.5 mM Na_2_EDTA. Samples were mixed with 2 μL of loading buffer and incubated at 45 °C for 2 min before being subjected to electrophoresis on 1% agarose gels in 100 mM Tris-borate (pH 8.3) and 2 mM EDTA. Gels were stained with 1 μg/mL ethidium bromide for 20 min and destained with distilled water for 10 min. DNA bands were visualized with medium-range ultraviolet light and quantified using an Alpha Innotech digital imaging system (Protein Simple). IC_50_ values (the concentration of drug required to inhibit enzyme activity by 50%) were calculated using GraphPad Prism Version 10.0.3 utilizing a nonlinear regression analysis with 95% confidence intervals.

### Topoisomerase IV-Catalyzed DNA Decatenation

DNA decatenation assays were based on previously published protocols.^22,73^ Reactions were performed in the absence or presence of increasing concentrations of OSUAB-0284, gepotidacin, and ciprofloxacin. Assay mixtures contained 100 nM wild-type (GrlA and GrlB) or 50 nM for mutant (GrlA^S81Y^ and GrlB) *B. anthracis* topoisomerase IV, 5 nM kDNA, 5 mM DTT and 1 mM ATP in 20 μL of 40 mM HEPES-KOH (pH 7.6), 25 mM NaCl, 100 mM KGlu, and 10 mM Mg­(OAc)_2_. In assays that examined the GrlA^E85K^ mutant *B. anthracis* topoisomerase IV, 100 nM enzyme (GrlA^E85K^ and GrlB) were used, and the ATP concentration was increased to 1.5 mM. Assay mixtures that contained wild-type and GrlA^E85K^ *B. anthracis* topoisomerase IV were incubated at 37 °C for 60 min, and assay mixtures that contained GrlA^S81Y^ were incubated at 37 °C for 20 min. Incubation times and conditions represent the minimum time required to decatenate >95% of the kDNA in the absence of drug for the respective enzymes. Reactions were stopped by the addition of 3 μL of a mixture of 0.77% SDS and 77.5 mM Na_2_EDTA, subjected to electrophoresis, and visualized as described for gyrase-catalyzed DNA supercoiling. IC_50_ values were calculated using GraphPad Prism Version 10.0.3 utilizing a nonlinear regression analysis with 95% confidence intervals.

### In Vivo Studies

In vivo studies were carried out in 6–8-week-old female BALB/c mice (Charles River, Kingston, NY). For in vivo dosing, OSUAB-0284 (as the methanesulfonate salt) was formulated in 20% hydroxypropyl-β-cyclodextrin in sterile water for injection. Ciprofloxacin for injection (2 mg/mL, Sagent Pharmaceuticals) was used as provided. Research was conducted under an Institutional Animal Care and Use Committee (IACUC) approved protocol in compliance with the Animal Welfare Act, Public Health Service Policy on Humane Care and Use of Laboratory Animals, and other federal statutes and regulations relating to animals and experiments involving animals. The facility where this research was conducted is accredited by the AAALAC International and adheres to the principles stated in The Guide for the Care and Use of Laboratory Animals, National Research Council, 2011. All in vivo study cohort sizes were supported by power analysis and are consistent with the established model. Mice were equally and randomly distributed into test groups upon the conclusion of each study. Investigators were not blinded to treatment identity and data analysis was appropriate for this proof-of-concept screen.

### Pharmacokinetics Study

A pharmacokinetics study was executed to inform dosing regimens of OSUAB-0284 in the *B. anthracis* infection model. Cohorts of mice (*n* = 3/time point) were given single doses of OSUAB-0284 (as the methanesulfonate salt) either by intravenous tail-vein injection (10 mg/kg) or oral gavage (10 or 50 mg/kg). Following dosing, mice were euthanized by barbiturate overdose at predetermined time points and terminal plasma samples were collected. Plasma concentrations of OSUAB-0284 were quantified using LC-MS/MS. Briefly, 1 mg/mL stock solutions of OSUAB-0284 and gepotidacin were prepared in 1:1 acetonitrile:water. A standard curve in plasma was prepared over a concentration range (10,000–1 ng/mL). Quality control samples were prepared at 10,000, 1,000, and 20 ng/mL. Separate stock solutions of OSUAB-0284 were used to make standard curve samples and quality control samples. Plasma samples (25 μL) were protein precipitated by addition of 75 μL acetonitrile containing 250 ng/mL gepotidacin as an internal standard. Samples were mixed and clarified by centrifugation prior to submission for LC-MS/MS. Antibacterial concentrations were quantified using a Sciex 5500 Triple Quad in line with a Shimadzu Prominence UPLC. The highest concentration of the standard curve for each was defined as the upper limit of quantification (ULOQ) and any sample above ULOQ was serially diluted into naïve matrix for reanalysis. All analytes were quantified in multireaction mode (MRM) using the following parameters: OSUAB-0284: 487.2 → 305.2, DP 206, CE 31, CXP 22; gepotidacin: 449.2 → 148.2, DP 206, CE 37, CXP 20. The source parameters of curtain gas, capillary voltage, temperature, GS1, and GS2 were 30, 2000 V, 550 °C, 30, and 40, respectively. Samples were separated using a HydroRP column (Phenomenex, 30 × 2 mm, 2.5 μm) under a gradient of acetonitrile +0.1% formic acid into water +0.1% formic acid over 4 min (T 0 min = 0% organic, 2.5 min = 40%, 3 min = 95%, 3.5 min = 95%, 3.7 min = 0%). The resulting plasma concentration data was analyzed using noncompartmental analysis (Phoenix 8.4, Certara).

### In Vivo Tolerability Study

The in vivo safety of orally administered OSUAB-0284 (as the methanesulfonate salt) was assessed in mice at 10, 50, and 100 mg/kg following a single dose or three consecutive days of dosing (q12h). OSUAB-0284 cohorts (*n* = 3/dose) were compared to a vehicle treated only group (*n* = 3). Animals were monitored throughout the dosing windows and for at least 3 days following cessation of treatment for cage-side clinical scoring.

### Aerosol Infection Model

Spores of the *B. anthracis* Ames strain were generated in Leighton–Doi sporulation medium and purified as previously described.^74–76^ Spores were washed with water and purified through Hypaque-76 (Nycomed, Inc.) before being washed three more times. Prior to the animal challenge, spores were heat shocked at 65 °C for 30 min and held on ice.

Mice were challenged with Ames spores as previously described.^75,76^ A mean inhaled dose of 103.6 × LD_50_ was administered to female BALB/c mice by whole-body aerosol. Aerosol was generated using a three-jet Collison nebulizer.^77^ All aerosol procedures were controlled and monitored using the Automated Bioaerosol Exposure System operating with a whole-body rodent exposure chamber.^78^ Integrated air samples were obtained from the chamber during each exposure using an all-glass AGI-30 impinger. Aerosolized bacterial concentrations were serially diluted and plated on sheep blood agar plates. The inhaled dose of *B. anthracis* spores, represented as colony-forming units (CFU)/mouse, was estimated using Guyton’s formula.^79^ Mice were equally and randomly distributed into test groups upon the conclusion of each aerosol run.

### Efficacy Assessment

To evaluate the in vivo protection afforded by OSUAB-0284, cohorts of aerosol challenged mice (*n* = 10/group) were treated with either saline (IP, q12h), ciprofloxacin (30 mg/kg, IP, q12h), or one of two dose levels of OSUAB-0284 (34.4 mg/kg or 100 mg/kg, PO, q12h). All treatments were initiated at 24 ± 1 h post challenge and continued for a total of 14 days. All animals were monitored at least twice daily during the treatment phase and at least once daily after. Survival was assessed over 30 days. Any moribund animals were humanely euthanized and counted as dead. After study termination, lungs were excised from five animals per surviving group, homogenized, and serially plated on sheep blood agar to determine bioburden. Aliquots of homogenate were also heat shocked (65 °C for 30 min) to determine remaining lung spore burden. Bioburden results were analyzed by one-way ANOVA with Dunnett’s multiple comparisons test comparing OSUAB-0284 results to the ciprofloxacin cohort.
